# Extraction Process Optimization, Structural Characteristics and Gut Microbiota Metabolism Regulatory Effect of *Suillus luteus* Alkali-Extracted Polysaccharide

**DOI:** 10.3390/foods15173157

**Published:** 2026-09-06

**Authors:** Xiao Han, Zhenjia Sun, Decheng Mao, Tian Wang, Wenjie Ding, Haiyu Ji, Yingyu Yang, Bing Han

**Affiliations:** 1 School of Life Sciences, Yantai University, Yantai 264005, China; hanxiao7721@163.com (X.H.); szj000925@163.com (Z.S.); decheng0313@163.com (D.M.); 2 Key Laboratory of Molecular Pharmacology and Drug Evaluation, Ministry of Education, Collaborative Innovation Center of Advanced Drug Delivery System and Biotech Drugs in Universities of Shandong, School of Pharmacy, Yantai University, Yantai 264005, China; bluewt2000@163.com; 3 College of Food Science and Engineering, Tianjin University of Science and Technology, Tianjin 300457, China; dingwenjie1120@163.com; 4 Xinjiang Yuanxiang Agricultural Technology Co., Ltd., Hetian 848000, China; yyy1995@163.com

**Keywords:** *S. luteus* alkali-extracted polysaccharide, structural characteristics, gut microbiota, untargeted metabolomics

## Abstract

Edible fungal polysaccharides have been recognized as low-toxic natural candidates for tumor adjuvant therapy by regulating gut microbiota and host metabolism. This study aimed to prepare an *S. luteus* alkali-extracted (pH = 9) polysaccharide (SLP9) via optimized extraction and systematically elucidate the anti-tumor effects based on polysaccharide structure, genus-level gut microbiota and fecal metabolomics. Structural characterization via high-performance gel permeation chromatography (HPGPC), gas chromatography-mass spectrometry (GC-MS) and Fourier-transform infrared spectroscopy (FTIR) verified that SLP9 possessed abundant β-glycosidic linkages with a Xyl:Man:Glc:Gal molar ratio of 0.52:1.00:0.81:0.57 and an average molecular weight of approximately 1.58 × 10^7^ Da. An in vivo anti-tumor experiment revealed that high-dose SLP9 exhibited obvious tumor inhibition with an inhibitory rate of 40.28% via restoring microbial diversity and selectively enriching the beneficial genus *Lactobacillus*, which might be relevant with activating three top significant KEGG pathways tropane, piperidine and pyridine alkaloid biosynthesis, arginine biosynthesis, alkaloid biosynthesis from ornithine, lysine and nicotinic acid. Collectively, this study systematically revealed the intrinsic correlations between the SLP9 extraction process, structural characteristics, targeted regulation of intestinal microbial metabolism and anti-tumor activity. These findings provide comprehensive theoretical support for using SLP9 as a new microecological regulator for tumor adjuvant therapy.

## 1. Introduction

*Suillus luteus* is an ectomycorrhizal fungus widely distributed in temperate forest regions commonly used for improving rhizosphere soil nutrients and accumulate heavy metals, and is also a traditional wild edible and medicinal mushroom in China [1,2]. *S. luteus* is rich in polysaccharide, protein, dietary fiber, vitamins and various bioactive secondary metabolites, among which polysaccharides are recognized as the core functional component with significant biological activities, including anti-tumor, anti-inflammatory and antioxidant effects [3,4,5]. The extraction process significantly influences the structural characteristics and biological activities of the polysaccharides [6]. Compared to conventional aqueous extraction, alkaline extraction generally yields higher polysaccharide recovery and may result in superior biological activity [7].

Malignant tumors represent a major global public health issue, posing a serious threat to human life and health [8]. The development and progression of tumors are closely associated with intestinal microbiome dysbiosis [9]. The reduced diversity and structural imbalance in the gut microbiota can lead to intestinal barrier damage, systemic chronic inflammation and immune dysfunction, thereby creating a favorable microenvironment for tumor growth and metastasis [10]. Tumor burden further disrupts the homeostasis of the gut microbiota, forming a vicious cycle of “tumor progression—microbiome disruption—immunosuppression.” Numerous important metabolites produced by gut microbes act as key signaling molecules regulating host immune responses, significantly influencing host health [11]. Therefore, targeting the modulation of gut microbiota composition and microbial metabolism has been a novel and safe cancer adjuvant strategy.

The biological functions of fungal polysaccharides are entirely determined by the structural characteristics, including molecular weight, monosaccharide composition, glycosidic linkage pattern and branching degree [12]. High-molecular-weight heteropolysaccharides with abundant branched chains cannot be digested and absorbed by digestive enzymes in the upper human gastrointestinal tract, so as to reach the colon intact and act as specific prebiotics for beneficial intestinal bacteria [13]. The structural characteristics of polysaccharides would directly govern their fermentation efficiency and selective regulatory capacity on gut microbiota [14]. Appropriate glycosidic linkages can markedly enrich probiotics such as *Lactobacillus*, optimize microbial metabolic pathways, boost the synthesis of short-chain fatty acids and further activate host anti-tumor immune responses [15,16]. Accordingly, the structural characteristics of polysaccharides were of vital importance for regulating gut microbiota metabolic activities and exerting biological effects.

In the present study, the *S. luteus* alkali-extracted polysaccharide was prepared after orthogonal experiment optimization, the primary structure was comprehensively characterized by HPGPC, FT-IR, SEM and GC-MS combined methylation analysis and the gut microbiota regulatory effects were evaluated via an S180 sarcoma tumor-bearing mouse model, aiming to elucidate the regulatory axis of “polysaccharide structure–gut microbiota metabolism–antitumor effect”. The research data can deliver theoretical guidance for developing SLP9 into high-value functional food raw materials and nutritional supplements for tumor adjuvant treatment.

## 2. Materials and Methods

### 2.1. Materials and Reagents

Dried fruit bodies of *S. luteus* used in this experiment were purchased from Rino Food Store (Heilongjiang, China), which were harvested in mid-autumn (September 2023) at the physiological maturity stage with fully developed sporophores, complete cap and stipe structure. SPF-grade Kunming mice were supplied by Jinan Pengyue Laboratory Animal Breeding Co., Ltd. (Shandong, China). Basic chemical raw materials including anhydrous ethanol, hydrochloric acid and sulfuric acid were supplied by Tianjin Jiangtian Chemical Technology Co., Ltd. (Tianjin, China). Monosaccharide standards and T-series dextran calibration standards (3000, 10,000, 70,000, 100,000 and 500,000 Da) were obtained from Beijing Solarbio Science & Technology Co., Ltd. (Beijing China). All other experimental chemicals met analytical-grade criteria.

### 2.2. Extraction Process Optimization of Crude S. luteus Alkali-Extracted Polysaccharides

Dried *S. luteus* fruit body powder was mixed with deionized water (solid–liquid ratio of 1:15~1:25 g/mL), and the suspension pH was adjusted to 9~11 using sodium hydroxide solution for heating extraction (60~80 °C). The sample was extracted twice, and the supernatants obtained from the two extractions were combined and concentrated by rotary evaporation. Anhydrous ethanol was added into the concentrated solution to achieve a final volume fraction of 80% for alcohol precipitation. The precipitates were harvested and re-dissolved in deionized water to obtain a crude polysaccharide solution. Polysaccharide yield was calculated as the ratio of crude polysaccharide weight to initial fungal powder weight, which was used as the evaluation indicator of orthogonal experiment [17].

### 2.3. Purification of SLP9

**Figure 1:**
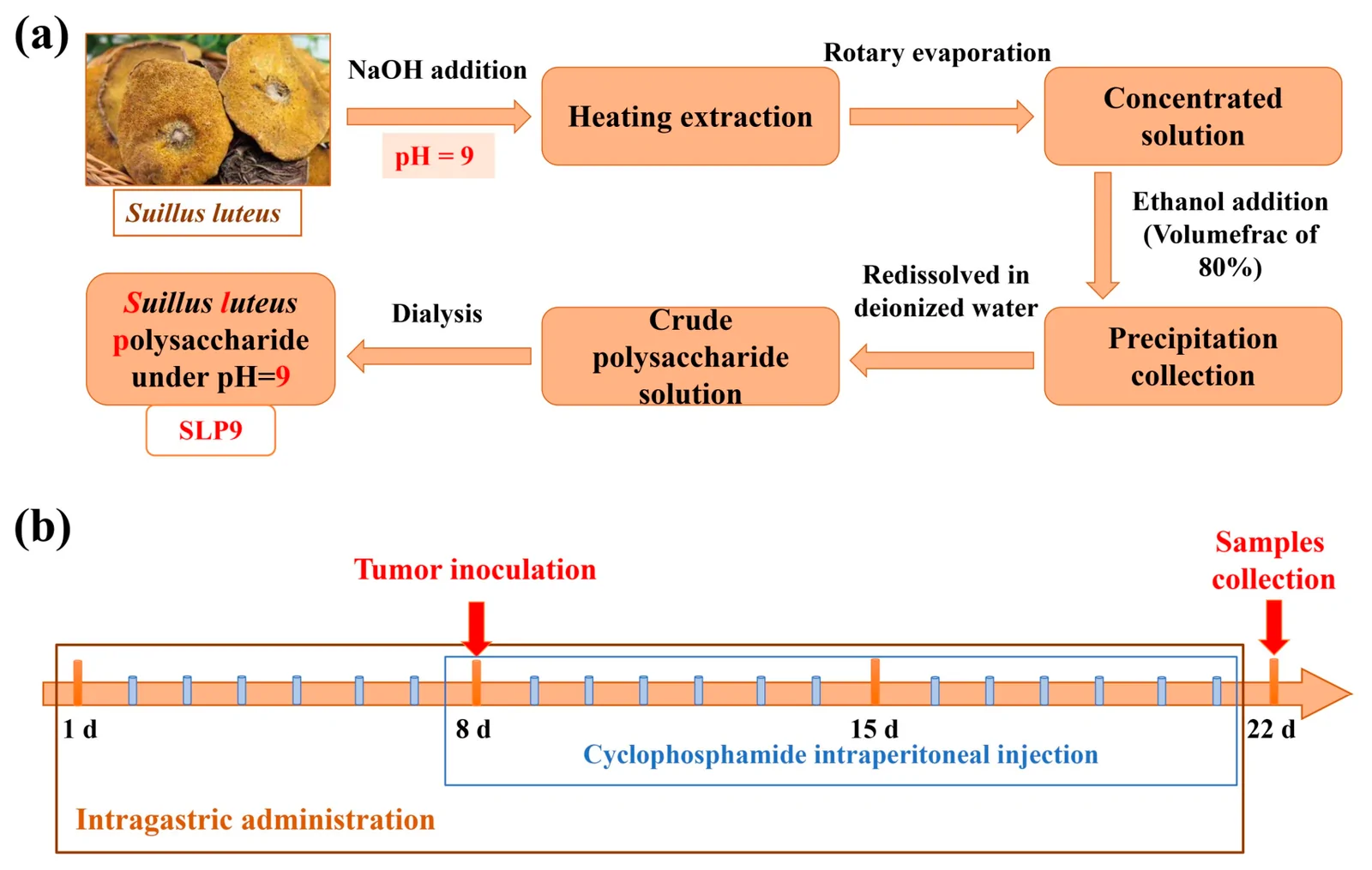
Schematic diagram of experimental procedures. (**a**) Extraction and purification workflow of SLP9; (**b**) animal experiment design.

The solution was dialyzed (molecular weight cut-off of 300 kDa) against deionized water to remove small molecular impurities. After vacuum freeze-drying, purified *S. luteus* polysaccharide (SLP9) was obtained. The whole extraction procedure is illustrated in Figure 1a.

### 2.4. Molecular Weight and Monosaccharide Composition Determination of SLP9

High-performance gel permeation chromatography (HPGPC, Agilent-1200 series, Santa Clara, CA, USA) was employed for the molecular weight distribution of SLP9. A dried sample was dissolved in deionized water (1 mg/mL) and filtered through a 0.45 μm aqueous membrane before injection. The molecular weight distribution was analyzed by HPGPC equipped with a TSK-gel G4000PWxl column (7.8 mm × 300 mm, Tosoh Bioscience, Tokyo, Japan) and a refractive index detector (Agilent, Santa Clara, CA, USA). Dextran standards were used to establish the molecular weight calibration curve. The average molecular weight of SLP9 was calculated according to the retention time [18].

Gas chromatography-mass spectrometry (GC-MS, Agilent 7000C/US1521U204, Santa Clara, CA, USA) was further applied to characterize the monosaccharide compositions of SLP9. A dried sample was hydrolyzed with trifluoroacetic acid. After neutralization and vacuum evaporation, the hydrolysate underwent oximation reaction with hydroxylamine hydrochloride in pyridine, followed by acetylation with acetic anhydride. The obtained derivatives were filtrated before injection [1].

### 2.5. Characteristic Functional Groups and Micromorphology Determination of SLP9

Fourier-transform infrared spectroscopy (FT-IR, Bruker VECTOR-22, Karlsruhe, Germany) was applied to characterize the functional groups of SLP9. A dried sample was fully ground and blended with KBr, then pressed into thin pellets. The spectrum was recorded within the wavenumber of 4000–400 cm^−1^. Functional group information of the polysaccharide was analyzed according to the positions of characteristic absorption bands [19].

Scanning electron microscopy (SEM, JEOL JSM-IT300LV, Tokyo, Japan) was used to determine the micromorphology of SLP9. The dried polysaccharide sample was fixed on conductive adhesive and subjected to gold-sputtering treatment under vacuum. The observation was carried out at an accelerating voltage of 10.0 kV, and micrographs were captured at magnifications of 100× and 500× to investigate the fragment structure and surface features [20].

### 2.6. Glycosidic Linkage Pattern Determination

The linkage pattern of SLP9 was determined via the methylation procedure. Dried polysaccharide was dissolved in anhydrous dimethyl sulfoxide. Basification was performed with sodium hydride under a nitrogen atmosphere, followed by permethylation with iodomethane. The permethylated product was extracted, washed and evaporated to dryness, and then was hydrolyzed with trifluoroacetic acid to release partially methylated monosaccharides, which were further reduced by sodium borohydride and acetylated with acetic anhydride to produce partially methylated alditol acetates (PMAAs), which were analyzed through GC-MS detection [21].

### 2.7. Animal Grouping and In Vivo Anti-Tumor Assay

The overall animal experimental schedule is illustrated in Figure 1b. A total of 50 specific pathogen-free (SPF) male Kunming mice weighing 25 ± 2 g were raised under specific pathogen-free surroundings. Animals were provided with an unlimited sterile diet and drinking water. The breeding environment was controlled at 22~25 °C with a relative humidity ranging from 40%~70%, accompanied by a 12 h light–dark circulation. All animal operation protocols gained authorization from the Ethics Committee of Tianjin University of Science and Technology (approval no.: 2023029; 16 November 2023). After one week of adaptive feeding, healthy mice were randomly divided into 5 groups using a random number table method to eliminate individual differences: blank group, model group, cyclophosphamide (CTX) positive control group, low-dose SLP9 group (SLP9-L, 100 mg/kg) and high-dose SLP9 group (SLP9-H, 200 mg/kg).

Mice in the blank, model and CTX groups received 0.2 mL normal saline by intragastric administration every day, while that in the SLP9-L and SLP9-H groups were intragastrically administered with corresponding concentrations of SLP9 daily. On day 8, all mice except those in the blank group were subcutaneously inoculated with 0.2 mL of an S180 tumor cell suspension (cell concentration: 1 × 10^7^ cells/mL) at the right axillary region to establish the subcutaneous tumor-bearing mouse model. For the positive control group, CTX was then injected intraperitoneally at a dosage of 25 mg/kg. All treatments lasted until day 21, and then the solid tumors of each group were collected for anti-tumor effect evaluation, with the calculation formula for tumor inhibitory rate (%) listed below: inhibitory rate (%) = (M_1_ − M_2_)/M_1_ × 100. The symbol M_1_ stands for the average tumor weight (g) of the model group, and M_2_ refers to the average tumor weight (g) of mice from various experimental treatment groups [22].

### 2.8. 16S rRNA Gene Amplicon Sequencing

Fresh fecal samples were collected from the blank, tumor model, CTX and SLP9-H groups for total genomic DNA extraction. DNA integrity and purity were assessed via 1% agarose gel electrophoresis. The V3–V4 hypervariable region of the bacterial 16S rRNA gene was PCR-amplified using barcode-labeled universal primers (338F: 5’-ACTCCTACGGGAGGCAGCAG-3’; 806R: 5’-GGACTACHVGGGTWTCTAAT-3’), with three technical replicates per sample. PCR products were purified by 2% agarose gel electrophoresis and quantified using the QuantiFluor™-ST blue fluorescence quantification system (Promega, Madison, WI, USA). Qualified amplicons were ligated to Y-type sequencing adapters and enriched by secondary PCR to construct sequencing libraries. The libraries were denatured with sodium hydroxide to generate single-stranded DNA templates and sequenced on the MiSeq platform (Illumina, San Diego, CA, USA) with the PE250 paired-end strategy. Raw sequencing reads were preprocessed using FASTP (v0.23.2) and FLASH (v1.2.11) with consistent filtering criteria. Briefly, low-quality terminal bases with a Phred score < 20 were trimmed via a 50 bp sliding window, and sequences with a window-averaged quality score < 20 were truncated. Reads shorter than 50 bp after trimming and sequences containing ambiguous N bases were discarded. Paired-end R1 and R2 reads were merged with a minimum overlap of 10 bp and a maximum overlap mismatch rate of 0.2. Valid sequences were demultiplexed and strand-corrected based on barcodes and primers, with zero barcode mismatches and a maximum of two primer mismatches permitted. High-quality clean sequences were processed for microbial bioinformatic analysis. Kraken2 (v2.1.2) was used to identify and remove host mouse genomic reads to eliminate host DNA contamination. The remaining microbial sequences were analyzed using QIIME 2 (v2023.9). The DADA2 plugin (v2022.2) was applied for sequence denoising, chimera removal and high-resolution amplicon sequence variant (ASV) table construction (replacing conventional OTU clustering). ASV taxonomic classification was performed against the SILVA 138 reference database. All raw FASTQ sequencing data were deposited in the NCBI SRA database. The final valid sequences were used for taxonomic annotation and quantitative analysis of intestinal microbial community composition and abundance [23].

### 2.9. Untargeted Metabolomics Analysis of Intestinal Metabolites

Fecal samples collected from the model and SLP9-H groups were fully ground under a liquid nitrogen environment. A total of 100 mg of ground fecal powder was mixed with 500 μL 80% methanol aqueous extract, followed by a 5 min ice bath static treatment. The mixture was centrifuged at 15,000× *g* and 4 °C for 20 min to acquire supernatant. A Dionex Ultimate 300 liquid chromatography instrument (Thermo Fisher Scientific, Germering, Germany) coupled with a C18 analytical column (2.1 mm × 100 mm, particle size 1.7 μm, Thermo Fisher, Carlsbad, CA, USA) was used to separate metabolite components. The UHPLC separation was performed with mobile phase A (0.1% formic acid in water) and mobile phase B (acetonitrile). The linear gradient elution was set as follows: 0–1 min, 95% A; 1–5 min, 95–40% A; 5–8 min, 40–0% A; 8–11 min, 0% A; 11–14 min, 0–40% A; 14–15 min, 40–95% A; and 15–18 min, 95% A. The flow rate was 0.2 mL/min. The Q Exactive™ mass spectrometer (Thermo Fisher Scientific, Bremen, Germany) was operated in both positive and negative polarity modes; spray voltage = 2.8 kV, capillary temperature = 320 °C, sheath gas flow = 40 arb and auxiliary gas flow = 10 arb. Pooled quality control (QC) samples were prepared by mixing equal volumes of supernatant from all fecal samples. One QC sample was injected every six experimental samples throughout the analytical sequence to monitor instrument stability, signal drift and method reproducibility. Raw UHPLC-MS/MS data were processed by TraceFinder 3.2.0 software for peak picking, peak alignment and metabolite quantification. Key processing parameters were set: retention time tolerance of 0.2 min, mass tolerance of 5 ppm and signal to noise ratio threshold of 3. Peak intensities were normalized to total spectral intensity. Metabolites were qualitatively annotated by matching experimental MS-MS/MS spectra against the mzCloud database. Peak area was applied for the relative quantitative calculation of metabolites. Differential metabolites were further annotated using KEGG, HMDB and LIPID-MAPS databases for subsequent metabolic pathway analysis. Two-tailed Student’s *t*-test was used for univariate analysis, and Benjamini–Hochberg FDR (false discovery rate) correction was performed to correct multiple-testing errors. VIP (variable importance in projection) values were generated from the PLS-DA model to evaluate each metabolite’s contribution to group discrimination. Metabolites with VIP > 1, FDR < 0.05 and fold change ≥ 2 or ≤0.5 were identified as differential metabolites [24].

### 2.10. Data Analysis

All experimental data were obtained from no less than three parallel replicates and displayed as mean ± standard deviation. Statistical comparisons were conducted via one-way analysis of variance on SPSS 26.0 statistical software. A threshold of *p* < 0.05 was defined as statistically significant, and marked as * or #. All visualized graphs were either exported directly from testing instruments or plotted with Microsoft Excel 2021 to intuitively reflect the variation tendencies and inter-group discrepancies.

## 3. Results

### 3.1. Optimal Extraction Process Parameter Analysis

An L_9_(3^3^) orthogonal array design was carried out with polysaccharide yield as the evaluation index. Three variables, extraction temperature, pH value and solid–liquid ratio, were investigated, and all experimental data were summarized in Table 1.

Range analysis revealed that the influencing sequence of tested factors on the yield of *S. luteus* polysaccharides followed the order extraction temperature (R = 1.52) > solid–liquid ratio (R = 0.29) > pH value (R = 0.21).

According to the mean k values of each factor level, the optimal level for extraction temperature, pH and solid–liquid ratio was 70 °C, pH = 10 and 1:25 g/mL, respectively. The optimal combination was therefore determined as an extraction temperature of 70 °C, pH of 10 and solid–liquid ratio of 1:25 (g/mL). This combination corresponded to Test No. 5, which achieved the maximum polysaccharide yield of 6.46%.

A variance analysis of the orthogonal test is presented in Table 2.

As presented, the extraction temperature exerted a significant influence on the yield of crude *S. luteus* polysaccharide (*p* < 0.05), while the pH value and solid–liquid ratio showed insignificant effects (*p* > 0.05). For industrialized production, economic efficiency was taken as a priority. For variables without significant influence on extraction yield, the most cost-efficient levels were adopted rather than the levels achieving maximum laboratory yield [25].

Consequently, the economical extraction parameters suitable for scale-up production were determined as follows: extraction temperature of 70 °C, pH = 9 and solid–liquid ratio of 1:15 (g/mL). Triplicate verification experiments were implemented to evaluate the practical extraction performance under the revised conditions. The average polysaccharide yields reached 6.32 ± 0.29%.

Under the optimal parameters, the crude polysaccharide solution obtained was purified by dialysis, and the resulting purified polysaccharide from the alkaline extract (pH = 9) of *S. luteus* was obtained, named SLP9. The total sugar (91.58 ± 2.73%) and protein contents (3.95 ± 0.18%) of SLP9 were determined through the phenol-sulfuric acid method and coomassie brilliant blue colorimetric method.

### 3.2. Molecular Weight and Monosaccharide Composition Analysis of SLP9

**Figure 2:**
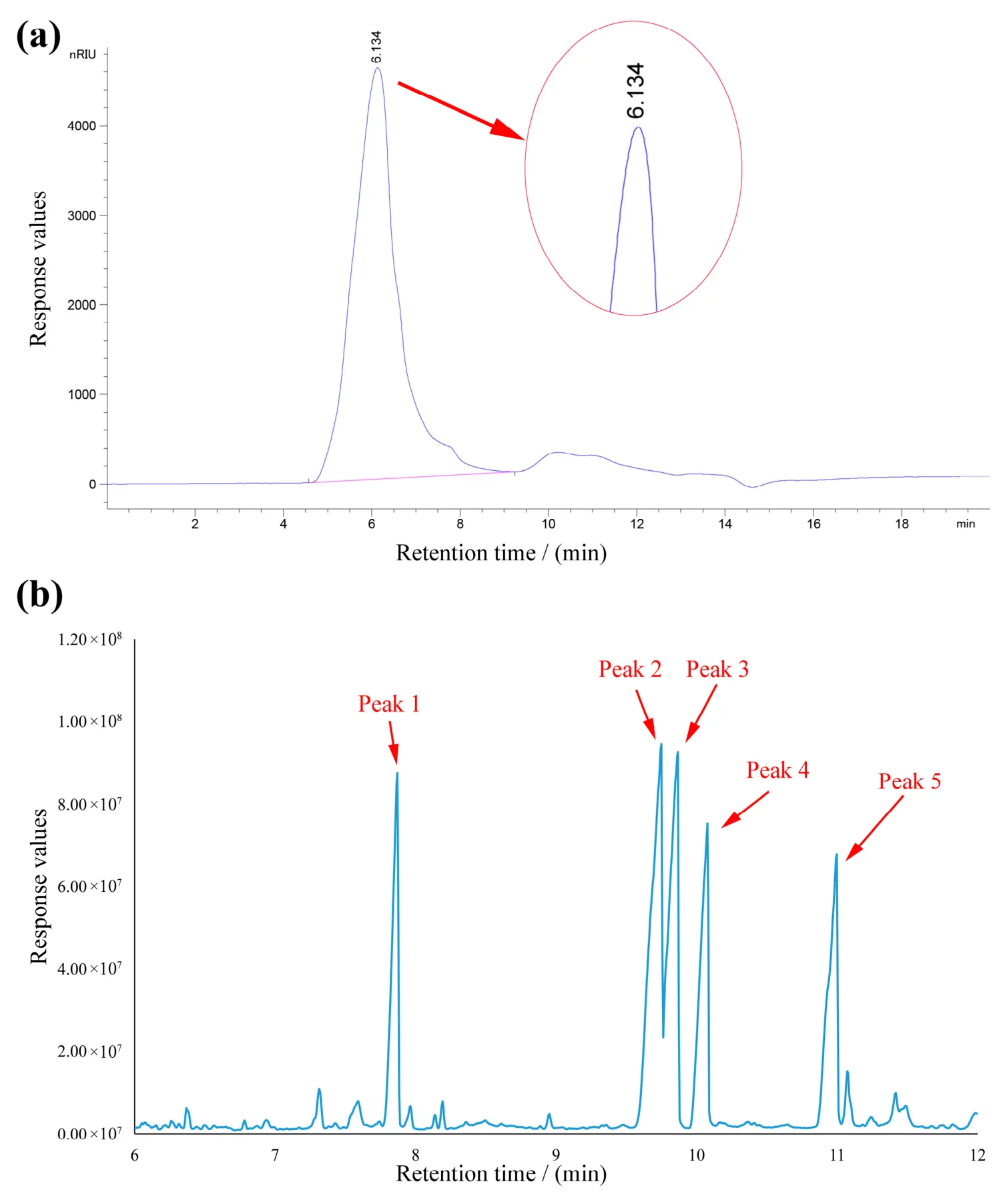
Chromatograms of SLP9. (**a**) HPGPC chromatogram for molecular weight distribution; (**b**) GC-MS total ion chromatogram of methylated derivatives.

Figure 2a shows the HPGPC profile of purified SLP9. The main peak appears at a retention time of 6.134 min, exhibiting a symmetric peak shape without obvious trailing or miscellaneous peaks, which indicated that the SLP9 possessed relatively concentrated molecular weight distribution and favorable homogeneity, and the purification procedure effectively removed heterogeneous polysaccharide impurities [26]. Combined with the standard calibration curve (y = −0.5827x + 10.7730, R^2^ = 0.9990; y = lg Mw, x = Retention time), the average molecular weight of SLP9 was calculated to be approximately 1.58 × 10^7^ Da.

Figure 2b displays the GC-MS total ion chromatogram of hydrolyzed SLP9 derivatives. Five well-resolved chromatographic peaks (Peak 1–Peak 5) were successfully separated, corresponding to D-Xylose (D-Xyl), D-Mannose (D-Man), D-Glucose (D-Glc), D-Galactose (D-Gal) and creatinine internal standard, respectively. The result exhibited that SLP9 was a kind of heteropolysaccharide with a Xyl:Man:Glc:Gal molar ratio of 0.52:1.00:0.81:0.57.

### 3.3. Characteristic Functional Groups and Apparent Morphology Analysis of SLP9

**Figure 3:**
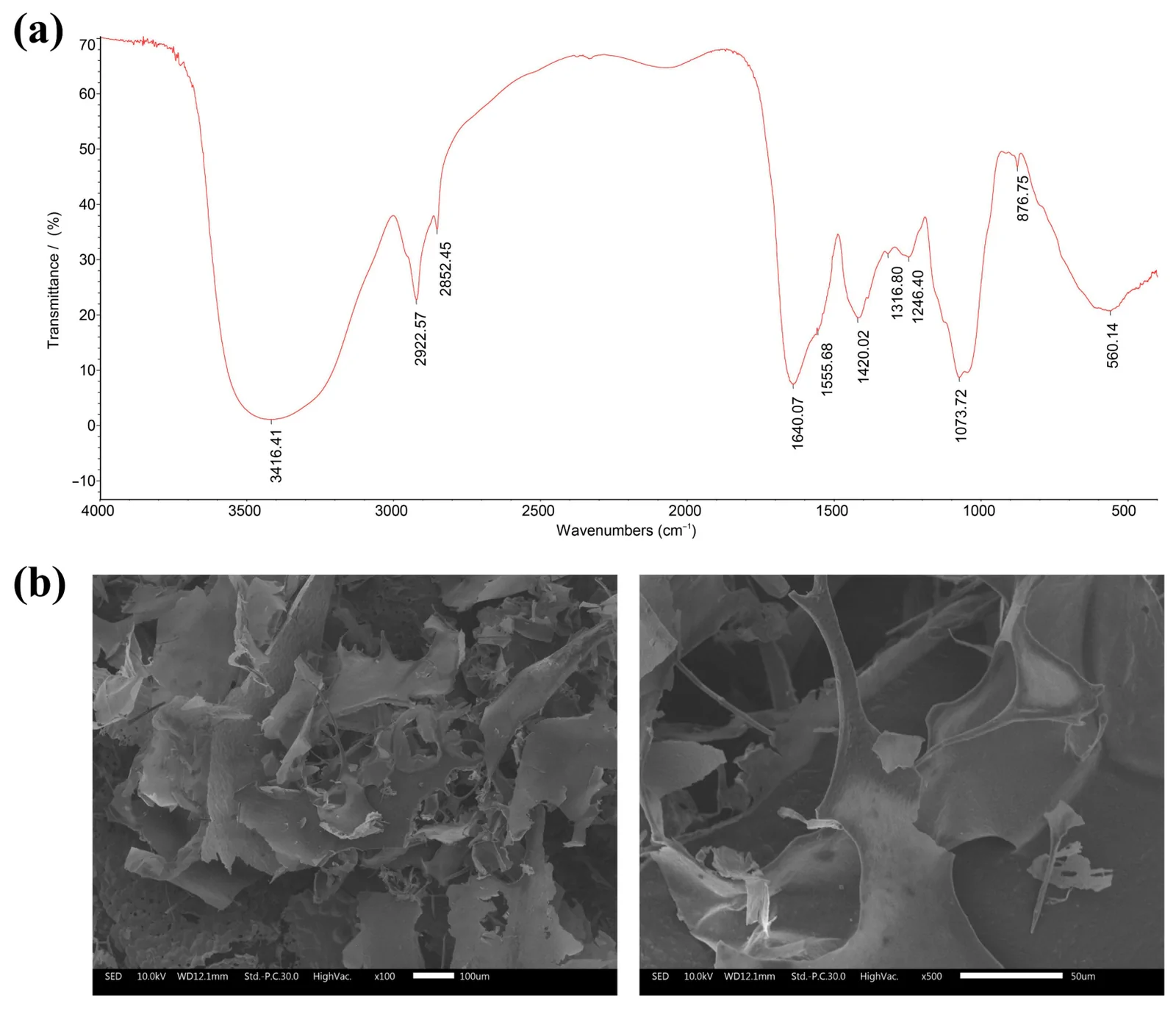
FTIR spectrum (**a**) and SEM images (**b**) of SLP9.

The FTIR spectrum of SLP9 is shown in Figure 3a. The broad and strong absorption peak at 3416.41 cm^−1^ was attributed to the stretching vibration of hydroxyl groups (-OH), and the peaks at 2922.57 cm^−1^ and 2852.45 cm^−1^ could be corresponded to C-H stretching vibrations of alkyl groups, which was the characteristic band of polysaccharides [27]. The absorption at 1640.07 cm^−1^ originated from the bending vibration of bound water and weak C=O stretching [28]. The peak at 1073.72 cm^−1^ reflected the stretching vibration of C-O-C glycosidic bonds on the pyranose ring [29]. Importantly, the characteristic band at 876.75 cm^−1^ indicated the existence of β-type glycosidic linkages, confirming that SLP9 belonged to pyranose polysaccharide [30].

SEM micrographs (Figure 3b) characterized the micromorphology of SLP9. At ×100 magnification, SLP9 exhibited irregular sheet-like fragments with non-uniform sizes. Under higher magnification (×500), the sheets displayed wrinkled and curled edges, and partial stacking was observed. The surface of slices was relatively smooth, revealing that the polysaccharide molecules aggregated to form layered structures after lyophilization.

### 3.4. Glycosidic Bond Configuration Analysis of SLP9

Methylation combined with GC-MS analysis identified ten types of partially methylated alditol acetates with diverse glycosidic linkages, and the result is presented in Table 3.

Among all residues, (1→4)-Xyl*p* exhibited the highest proportion (23.22%), followed by terminal (1→)-Glc*p* (13.61%), branched (1→3,6)-Man*p* (12.53%) and (1→6)-Man*p* (11.11%). Three kinds of double-substituted branched residues, namely (1→3,6)-Manp, (1→4,6)-Glcp and (1→2,6)-Manp, were detected, accounting for 22.93% in total, which revealed that SLP9 possessed a relatively high degree of branching [31]. The residue types detected by methylation were consistent with monosaccharide composition results.

### 3.5. Anti-Tumor Effect of SLP9

**Figure 4:**
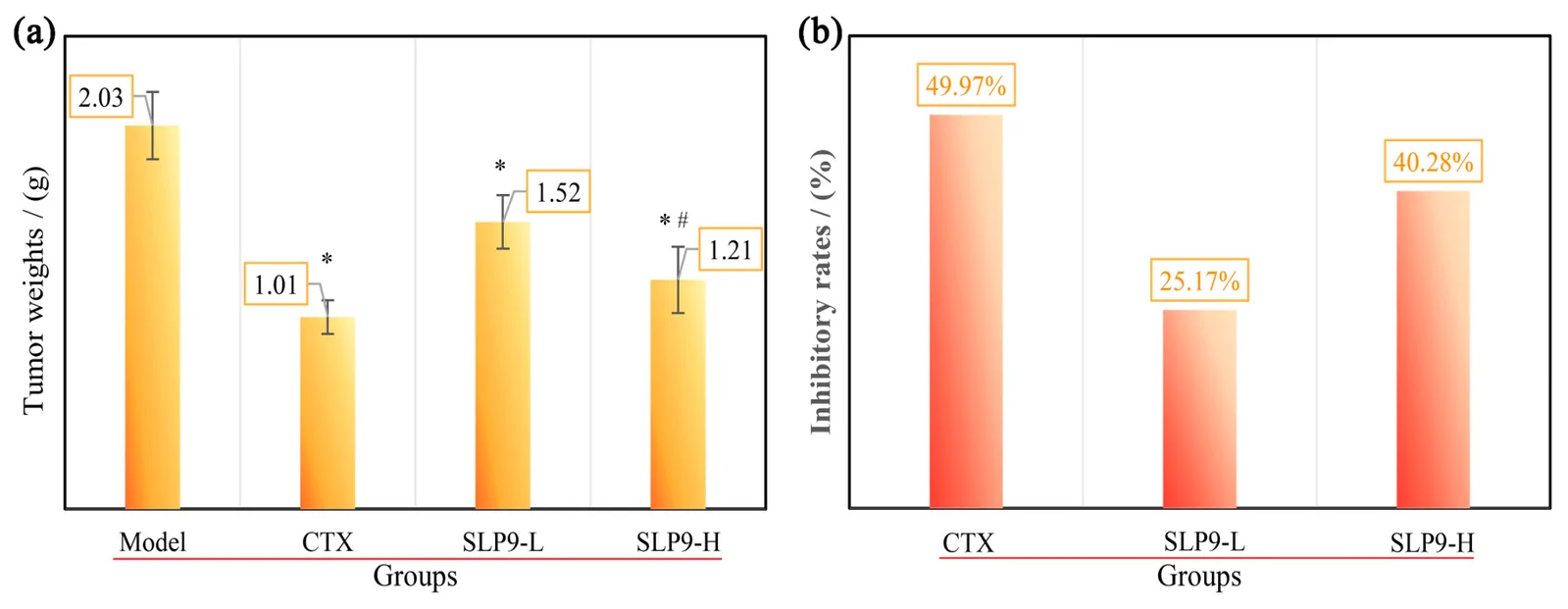
Tumor weights (**a**) and inhibitory rates (**b**) of different treatment groups (*n* = 8). Note: *, significant difference at *p* < 0.05 compared with the model group; #, significant difference at *p* < 0.05 compared with the SLP9-L group.

The tumor weights of mice in each group are presented in Figure 4a. The average tumor weight of the model group was 2.03 g. After CTX treatment, the average tumor weight was markedly reduced to 1.01 g (*p* < 0.05). For SLP9-treated groups, the average tumor weights were 1.52 g in the SLP9-L group and 1.21 g in the SLP9-H group, indicating that SLP9 could suppress the growth of S180 solid tumors in a dose-dependent manner. The tumor inhibitory rates are shown in Figure 4b. The CTX group exhibited the highest inhibitory rate of 49.97%. The tumor inhibition rate of SLP9-L group was 25.17%, while the rate increased to 40.28% in the SLP9-H group. These data confirmed that SLP9 possessed obvious anti-tumor effect in vivo, and the inhibitory effect was enhanced with an increase in dosage [32], but inferior to the positive drug CTX.

### 3.6. Effects of SLP9 Treatment on the Gut Microbiota Compositions

**Figure 5:**
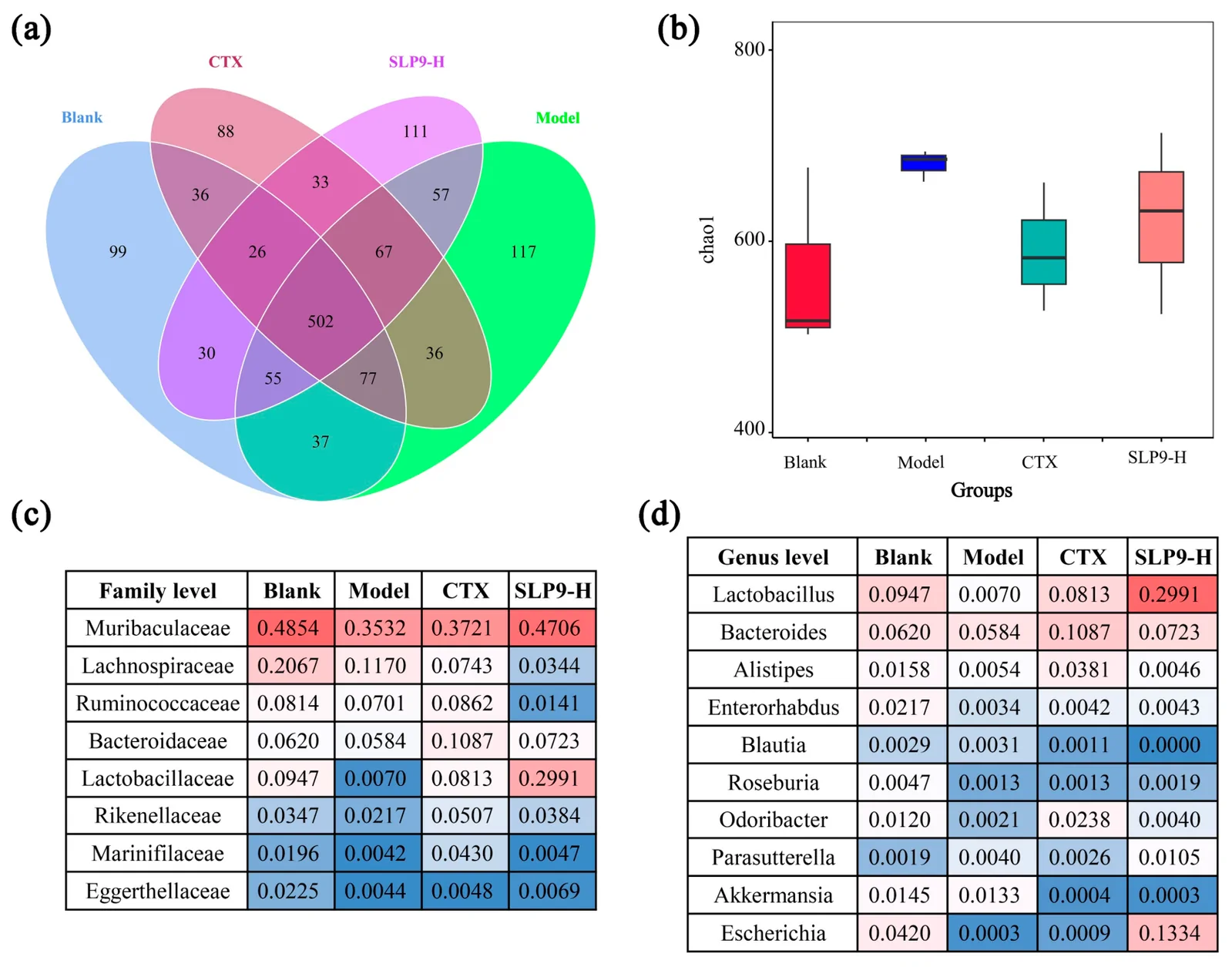
Analysis of gut microbiota in mice. (**a**) Venn diagram of OTUs; (**b**) Chao1 index; (**c**) divergent microbiota at the family level; and (**d**) divergent microbiota at the genus level (*n* = 3).

Fecal microbial genomic DNA was extracted for high-throughput sequencing of the 16S rRNA gene, and the relevant results are shown in Figure 5.

The Venn diagram (Figure 5a) revealed 502 shared OTUs across all groups. The unique OTUs of the blank, model, CTX and SLP9-H groups were 99, 117, 88 and 111, respectively, demonstrating that tumor inoculation and drug intervention reshaped gut microbial compositions. The Chao1 index (Figure 5b) reflected microbial community richness. The model group exhibited higher Chao1 value than the blank group. CTX treatment reduced microbial richness, while SLP9-H supplementation increased the Chao1 index and restored the gut microbiota species richness of tumor-bearing mice [33].

The group-specific differential taxa was analyzed [34]. At the family level (Figure 5c), Muribaculaceae and Lactobacillaceae were biomarker taxa enriched in the SLP9-H group. Tumor modeling reduced the abundances of Muribaculaceae, Lachnospiraceae and Lactobacillaceae. Compared with the model group, SLP9-H showed superior capacity to recover these beneficial families. At the genus level (Figure 5d), *Lactobacillus* was the most prominent biomarker of the SLP9-H group. The abundance was drastically decreased in the model group and greatly recovered after SLP9-H administration. Meanwhile, *Escherichia* was enriched in the SLP9-H group, and obvious variations in *Bacteroides*, *Alistipes*, *Roseburia* were observed among experimental groups.

Therefore, S180 tumor challenge disrupted intestinal microbial homeostasis. SLP9-H remodeled gut microbiota, elevated community richness and selectively enriched beneficial bacteria represented by *Lactobacillus*, which was possibly associated with the anti-tumor activity in vivo [35].

### 3.7. Effects of SLP9 Treatment on the Gut Microbiota Metabolites

Untargeted metabolomics analysis of fecal samples was performed to screen differential metabolites between the SLP9-H group and model group, and metabolites with *p* < 0.05 are summarized in Table 4. All differential compounds were divided into down-regulated and up-regulated groups according to log2FC values.

Overall, a greater number of metabolites showed significant down-regulation after SLP9 intervention, mainly covering glycerophospholipids, carnitines (ACar), prostaglandins, steroids, short peptides, alkaloids, toxins and some lipid derivatives.

(1) Glycerophospholipids: Large amounts of phosphatidylcholine (PC), phosphatidylinositol (LPC), lysophosphatidylethanolamine (LysoPE) and sphingolipid substances were significantly decreased, indicating that SLP9 intervention selectively could reshape intestinal lipid metabolism homeostasis, alleviate the abnormal accumulation of intestinal phospholipids related to inflammation and exert a protective effect on the intestine [36].
(2) Carnitine derivatives ACar: ACar 18:3 and ACar 20:2 expressions were significantly decreased. Carnitine was involved in fatty acid transport, and the content decrease suggested changes in the level of intestinal fatty acid transport and oxidation metabolism [37].
(3) Inflammatory mediators: 13,14-dihydro-15-ketoprostaglandin E2, corticosterone and other pro-inflammatory and stress-related metabolites significantly decreased, reflecting that SLP9 can alleviate intestinal inflammation and the stress state of tumor-bearing mice [38].
(4) Toxins, harmful secondary metabolites: vomitoxin, stercobilin, piperine, ecgonine (cocaine derivative) and its methyl ester were all significantly down-regulated, suggesting that SLP9 can reduce the accumulation of toxic and irritating metabolic products in the intestine [39].

Meanwhile, the metabolites that significantly increased after SLP9-H treatment mainly include bile acids, free amino acids, unsaturated carnitines, triterpenoids, adenine, lipids soluble in blood and plant-derived active derivatives.

(1) Bile acids: Dehydrocholic acid and chenodeoxycholic acid were significantly up-regulated. As vital signaling mediators governing microbiota homeostasis and intestinal mucosal integrity, elevated bile acids driven by SLP9 supplementation effectively remodel disordered gut microecology in tumor-bearing hosts and mitigate intestinal barrier injury [40].
(2) Amino acids and nucleotides: DL-Arginine, L-Arginine and adenine were prominently enriched. Arginine serves as a core substrate for systemic immune modulation and nitric oxide biosynthesis, whose accumulation substantially restores compromised anti-tumor immune capacity in S180 tumor-bearing mice [41].
(3) Triterpenoids, glycyrrhetinic acid and lactone-type active substances: Multiple triterpenoid and steroidal metabolites including 18-glycyrrhetinic acid, hydroxyursolic acid, hydroxymethylsterone and osthole glycosides were drastically up-regulated. Such compounds possess intrinsic anti-inflammatory and anti-proliferative properties, which collaboratively alleviate chronic intestinal inflammation and suppress tumor progression [42].

Apart from the above characterized functional metabolites, numerous differential metabolites with unclear physiological roles in tumor-bearing mice were also identified in this metabolomic profiling. The expression variations of these unannotated metabolites would lay a solid preliminary data foundation and candidate targets for follow-up mechanistic exploration and functional verification research.

In summary, SLP9 reshaped the fecal metabolic profile of S180 tumor-bearing mice: it suppressed the accumulation of pro-inflammatory lipids and harmful toxic metabolites, and elevated the levels of beneficial bile acids, immunomodulatory amino acids and microbial aromatic products. Combined with 16S rRNA gene sequencing results, the synergistic alteration of gut microbiota and fecal metabolites mediated by SLP9 might be the underlying mechanism for its intestinal protection and anti-tumor effects in vivo.

### 3.8. Effects of SLP9 Treatment on the Metabolic Pathways

**Figure 6:**
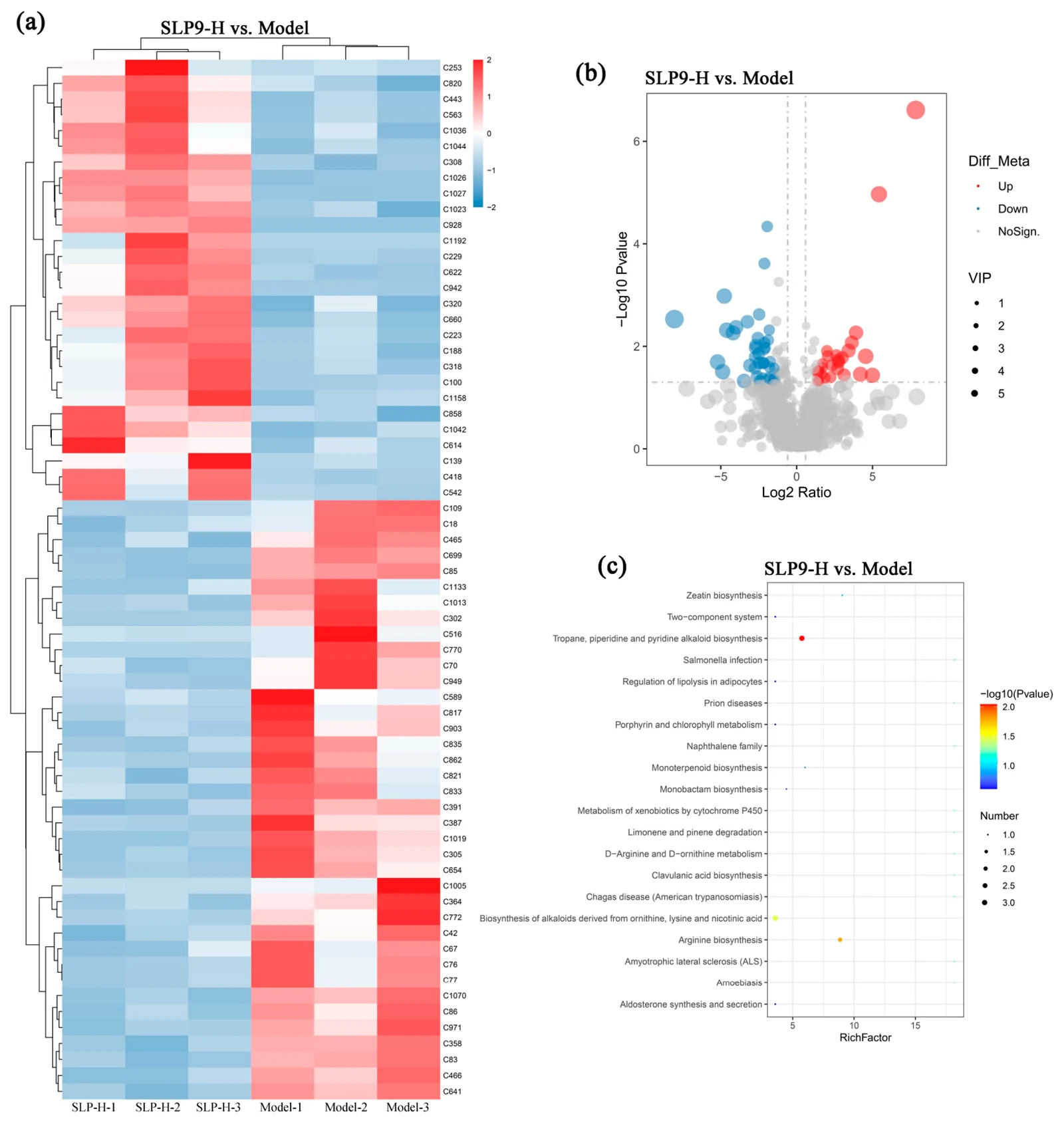
Fecal metabolomics analysis between the SLP9-H group and model group (*n* = 3). (**a**) Hierarchical clustering heatmap of differential metabolites; (**b**) volcano plot of differential metabolites; and (**c**) bubble plot of KEGG pathway enrichment.

After the SLP9 intervention treatment, the expressions of differential metabolites in tumor-bearing mice were illustrated in Figure 6.

Figure 6a presented the hierarchical clustering heatmap of differential metabolites. A red color indicated higher relative abundance of metabolites, while a blue color represents lower relative abundance; the color depth reflects the magnitude of abundance variation. Figure 6b showed the volcano plot for differential metabolites. Red dots correspond to significantly up-regulated metabolites, blue dots correspond to significantly down-regulated metabolites and gray dots represent metabolites without significant difference. The size of each dot indicates the VIP (variable importance in projection) value, and larger dots mean metabolites with a higher contribution to distinguishing two groups. A total of 66 significantly differential metabolites were identified in the SLP9-H group versus model group, including 28 significantly up-regulated metabolites and 38 significantly down-regulated metabolites, which demonstrated that SLP9 treatment extensively remodeled the expression level of intestinal metabolites in tumor-bearing mice.

A bubble diagram of KEGG pathway enrichment is exhibited in Figure 6c. As shown, the horizontal axis represented the rich factor, and a higher value indicated stronger enrichment intensity. The vertical axis listed all enriched pathway names. Bubble color shifts gradually from blue to red with increasing −log10 (*p* value); a deeper red color corresponded to higher statistical significance of enrichment. Bubble size reflected the quantity of differential metabolites enriched in each pathway, and larger bubbles contained more differential metabolites. The significance order of the top three pathways from high to low was tropane, piperidine and pyridine alkaloid biosynthesis (mediated by C465 ecgonine, C466 ecgonine methyl ester and C903 piperine, *p* = 0.0090); arginine biosynthesis (mediated by C387 citrulline and C563 L-Arginine, *p* = 0.0157); biosynthesis of alkaloids derived from ornithine, lysine and nicotinic acid (mediated by C466 ecgonine methyl ester, C563 L-Arginine and C903 piperine, *p* = 0.0331).

Among all annotated KEGG pathways, tropane, piperidine and pyridine alkaloid biosynthesis showed the most prominent enrichment. The corresponding differential metabolites are nitrogen-containing heterocyclic alkaloids such as hyoscyamine and nicotine derivatives, which exert comprehensive anti-inflammatory, antioxidant and immunomodulatory effects. Notably, ornithine, lysine and nicotinic acid serve as core biosynthetic precursors specifically driving the synthetic cascades of pyrrolidine, piperidine and pyridine alkaloid subfamilies, laying a material basis for the massive accumulation of these bioactive alkaloids after SLP9 intervention [43,44]. Significant enrichment indicated that SLP9 supplementation could stimulate gut microbiota to accumulate abundant bioactive alkaloids, alleviating chronic intestinal inflammation and stabilizing intestinal microecology in tumor-bearing mice.

As an indispensable immunomodulatory amino acid with potent anti-tumor bioactivity, arginine facilitates macrophage polarization and nitric oxide synthesis, thereby reversing a tumor-associated immunosuppressive microenvironment [41,45]. The striking up-regulation of the arginine biosynthetic pathway observed in the SLP9-H group was relevant with its in vivo tumor-suppressive efficacy and the remarkable enrichment of beneficial intestinal genera.

Consequently, SLP9 simultaneously activated amino acid metabolism and multiple alkaloid synthetic pathways. Arginine supplied precursors for alkaloid production, and abundant flora-derived bioactive alkaloids accumulated jointly to relieve intestinal inflammation and boost host immunity, providing insights into the plausible metabolic mechanism by which SLP9 modulates intestinal microecology and mediates anti-tumor activities.

## 4. Discussions

### 4.1. Extraction, Polysaccharide Structure and Structure–Activity Relationship

Numerous studies have verified that extraction procedures determine the molecular weight, monosaccharide composition, glycosidic linkage patterns and branching degree of fungal polysaccharides, and these structural traits are fundamental to their prebiotic and immunomodulatory capacities [46]. Compared with ordinary deionized water, alkaline electrolyzed water (pH = 12), which mainly consisted of hydroxide ions, was used to extract polysaccharides from *Codonopsis pilosula* and generated components with enhanced anti-tumor biological activity [46]. Accordingly, SLP9 was prepared by mild alkaline extraction at pH = 9 in the present study, which contained abundant β-glycosidic linkages with a monosaccharide molar ratio of Xyl:Man:Glc:Gal = 0.52:1.00:0.81:0.57 and an average molecular weight of approximately 1.58 × 10^7^ Da, and the tumor inhibition rate rose from 25.17% in the low-dose group (SLP9-L) to 40.28% in the high-dose group (SLP9-H). However, for *Suillus luteus*, polysaccharides isolated via conventional hot water extraction (average molecular weight of approximately 1.90 × 10^6^ Da with Fuc, Man, Glc and Gal molar ratio of 0.37:1.00:0.72:0.54) and acid extraction (average molecular weight of approximately 1.76 × 10^7^ Da with Xyl, Man, Glc and Gal molar ratio of 0.19:1.00:0.72:0.53) exerted stronger anti-tumor effects in tumor-bearing mice, with tumor inhibition rates reaching 53.14% and 61.14%, respectively [1,22], indicating that the high content of xylose and the excessively high molecular weight might be detrimental to the exertion of biological activity. Collectively, these findings indicated that alkaline extraction conditions exert adverse impacts on the biological activity of fungal polysaccharides derived from this strain.

### 4.2. Structural-Dependent Modulation of the Microbiota–Metabolite Regulatory Axis

Due to the resistance of non-starch fungal polysaccharides to digestive enzyme degradation in the upper gastrointestinal tract, they can reach the colon and mediate the metabolic remodeling of the intestinal microbiota, further exerting diverse systemic biological activities [47]. Accumulating microbiological evidence reveals that non-digestible fungal polysaccharides serve as selective carbon sources driving the enrichment of advantageous gut genera, and polysaccharide glycosidic linkages and side-chain monosaccharides govern their microbial selectivity [48]. *Lactobacillus* strains are commensal lactic acid bacteria with excellent gastrointestinal tolerance, inherent immunomodulatory and anti-tumor activity, which could remodel the gut tumor microenvironment and boost immunotherapy efficacy [49]. Consistent with previous reports, the genus-level analysis in this work identified drastically enriched *Lactobacillus* as a signature biomarker in the SLP9-H group; by contrast, the model group displayed *Lactobacillus* depletion and dysregulated microecology as a typical phenotype of S180 tumor-bearing mice. Combined with the expression profiles of differential metabolites, it could be inferred that SLP9 intervention could significantly enrich intestinal *Lactobacillus*, optimize the intestinal microecological environment, alleviate inflammatory responses and improve immune capacity, which might be relevant with the in vivo anti-tumor effect.

### 4.3. Correlation Analysis of Alkaloid, Arginine Metabolism and Anti-Tumor Activity

As vital secondary metabolites, alkaloids are involved in cell proliferation modulation, oxidative stress balance and host immune response, exhibiting potential capacities to suppress tumor cell proliferation and trigger tumor cell apoptosis [50]. Arginine is a pivotal functional amino acid governing anti-tumor immunity, and serves as a nitrogen source fueling tumor cell proliferation, meanwhile supporting the activation, expansion and effector molecule secretion of T lymphocytes and macrophages, and the metabolic reprogramming directly shaped the magnitude of host anti-tumor immune responses [51]. Collectively, SLP9-triggered metabolic reprogramming could optimize arginine turnover to promote the differentiation and function of immune cells, and collaborated with bioactive alkaloid metabolites to strengthen anti-tumor immune surveillance and restrain tumor proliferation, thereby mediating the favorable anti-tumor bioactivity.

## 5. Conclusions

In this study, an *S. luteus* heteropolysaccharide SLP9 was obtained under a weak base condition of pH = 9, which mainly contained abundant β-glycosidic linkages with a Xyl:Man:Glc:Gal molar ratio of 0.52:1.00:0.81:0.57 and average molecular weight of approximately 1.58 × 10^7^ Da. Animal experiment results demonstrated that SLP9 exhibited dose-dependent anti-tumor effects, achieving a tumor inhibition rate of 40.28% at high dosage, remodeled disturbed gut microbiota in S180 tumor-bearing mice and specifically enriched *Lactobacillus*, and mediated the significant KEGG pathways covering arginine and alkaloid biosynthesis. Collectively, this work clarified the potential connection among extraction process, polysaccharide structure, gut microbiota metabolism and anti-tumor capacity, supplying multi-dimensional theoretical support for developing edible fungal polysaccharides as microecology-targeted adjuvant anti-tumor candidates.

## Figures and Tables

**Table 1:** Orthogonal experiment results for crude *S. luteus* polysaccharides.

Factors	Extraction Temperature /(°C)	pH Value	Solid–Liquid Ratio /(g/mL)	Polysaccharide Yields /(%)
No. 1	60	9	1:15	4.45
No. 2	60	10	1:20	4.63
No. 3	60	11	1:25	4.57
No. 4	70	9	1:20	5.92
No. 5	70	10	1:25	6.46
No. 6	70	11	1:15	5.83
No. 7	80	9	1:25	4.71
No. 8	80	10	1:15	4.60
No. 9	80	11	1:20	4.67
k_1_	4.55	5.03	4.96	
k_2_	6.07	5.23	5.07	
k_3_	4.66	5.02	5.25	
Range value	1.52	0.21	0.29	

Note: k_1_, k_2_ and k_3_ represent the average polysaccharide yield of extraction temperature, pH value and solid–liquid ratio, respectively.

**Table 2:** Orthogonal experiment variance analysis for *S. luteus* polysaccharides.

Factors	Sum of Squares	df	F Values	Critical F Value	Significance
Extraction temperature	4.31	2	93.72	19.00	*
pH value	0.08	2	1.83	19.00	ns
Solid–liquid ratio	0.13	2	2.72	19.00	ns
Error	0.05	2			

Note: *, significant difference at *p* < 0.05; ns, no significant difference at *p* > 0.05.

**Table 3:** Methylation results of SLP9.

No.	Glycosidic Bonds	Retention Time/(min)	Sugar Residues	Ion Fragments	Proportions
					(%)
1	(1→)-Glc*p*	5.608	2,3,4,6-Me_4_	43,71,87,101,117,129,145,161,205	13.61%
2	(1→)-Gal*p*	6.424	2,3,4,6-Me_4_	43,71,87,101,117,129,145,161,205	8.45%
3	(1→6)-Glc*p*	6.510	2,3,4-Me_3_	43,58,71,87,101,117,129,145,161	6.27%
4	(1→4)-Xyl*p*	6.714	2,3-Me_2_	43,71,85,87,99,117,129,145,161	23.22%
5	(1→6)-Gal*p*	7.023	2,3,4-Me_3_	43,58,87,99,101,117,129,145,173	9.74%
6	(1→4)-Glc*p*	7.184	2,3,6-Me_3_	43,71,87,101,117,129,145,161,205	4.67%
7	(1→6)-Man*p*	7.308	2,3,4-Me_3_	43,58,71,87,101,117,129,145,161	11.11%
8	(1→3,6)-Man*p*	7.753	2,4-Me_2_	43,71,87,99,117,129,159,201,259	12.53%
9	(1→4,6)-Glc*p*	7.895	2,3-Me_2_	43,71,87,101,117,127,159,161,201	4.42%
10	(1→2,6)-Man*p*	8.025	3,4-Me_2_	43,71,87,99,117,129,159,189,231,261	5.98%

**Table 4:** Differential metabolite information in the SLP9-H group compared with the model group (*n* = 3).

Metabolites ID	Description	Formula	Retention Time/(min)	*m*/*z*	log2FC	*p* Values	Up or Down
C1005	Tributyrin	C_26_H_45_NO_5_S	5.90	301.17	−5.2129	0.0202	Down
C1013	Uridine	C_9_H_12_N_2_O_6_	0.58	243.06	−2.7210	0.0107	Down
C1019	Vomitoxin	C_15_H_20_O_6_	0.52	295.12	−4.1893	0.0055	Down
C1070	Aspartylphenylalanine	C_13_H_16_N_2_O_5_	3.83	281.11	−2.5567	0.0069	Down
C109	2-(1H-1,2,3-benzotriazol-1-yl)-N-(2,3-dihydro-1H-inden-2-yl)acetamide	C_17_H_16_N_4_O	13.14	293.14	−2.2638	0.0419	Down
C1133	4-morpholino-6-phenylthieno [2,3-d]pyrimidine	C_16_H_15_N_3_OS	2.89	298.10	−1.8128	0.0395	Down
C18	(3beta,9xi)-3-(beta-D-Glucopyranosyloxy)-14-hydroxycard-20(22)-enolide	C_29_H_44_O_9_	6.87	537.31	−1.4256	0.0454	Down
C302	ACar 18:3	C_25_H_44_NO_4_	7.06	423.33	−4.7685	0.0010	Down
C305	ACar 20:2	C_27_H_50_NO_4_	9.36	453.38	−2.4711	0.0024	Down
C358	Cafestol	C_20_H_28_O_3_	0.57	317.21	−2.4180	0.0479	Down
C364	Carvone	C_10_H_14_O	13.02	151.11	−2.0828	0.0106	Down
C387	Citrulline	C_6_H_13_N_3_O_3_	10.78	176.10	−3.2443	0.0033	Down
C391	Corticosterone	C_21_H_30_O_4_	3.25	347.22	−1.8710	0.0076	Down
C42	1-(3,4-dimethoxyphenyl)ethan-1-one oxime	C_10_H_13_NO_3_	3.50	196.10	−1.7900	0.0254	Down
C465	Ecgonine	C_9_H_15_NO_3_	3.25	186.11	−1.7418	0.0197	Down
C466	Ecgonine methyl ester	C_10_H_17_NO_3_	3.83	200.13	−2.1366	0.0114	Down
C516	GNH	C_12_H_18_N_6_O_5_	0.57	327.14	−4.8763	0.0312	Down
C589	LPC 14:0	C_22_H_46_NO_7_P	8.60	466.29	−3.4634	0.0474	Down
C641	LysoPC(22:0)	C_30_H_62_NO_7_P	13.20	580.43	−2.4512	0.0211	Down
C654	LysoPE(0:0/18:4(6Z,9Z,12Z,15Z))	C_23_H_40_NO_7_P	6.38	474.26	−4.6066	0.0048	Down
C67	11-Oxoetiocholanolone	C_19_H_28_O_3_	13.87	305.21	−1.4927	0.0424	Down
C699	N2-ethyl-N4-isopropyl-6-(methylthio)-1,3,5-triazine-2,4-diamine	C_9_H_17_N_5_S	11.23	228.13	−1.9400	0.0001	Down
C70	12-Methyltetradecanoic acid	C_15_H_30_O_2_	2.08	241.22	−2.5965	0.0393	Down
C76	13,14-dihydro-15-keto Prostaglandin E2	C_20_H_32_O_5_	8.14	351.22	−2.1976	0.0215	Down
C77	13,14-Dihydro-15-keto-PGE2	C_48_H_87_NO_8_P	9.25	351.22	−2.1712	0.0210	Down
C770	PC (15:0/16:0)	C_39_H_78_NO_8_P	14.09	720.55	−8.0549	0.0029	Down
C772	PC (16:0/17:0)	C_41_H_82_NO_8_P	8.25	748.59	−3.9912	0.0042	Down
C817	PC (18:4e/4:0)	C_30_H_54_NO_7_P	7.25	572.37	−2.7478	0.0266	Down
C821	PC (18:5e/20:5)	C_46_H_74_NO_7_P	9.60	784.53	−1.5626	0.0468	Down
C83	16,16-Dimethyl prostaglandin A1	C_22_H_36_O_4_	7.06	365.27	−2.5004	0.0140	Down
C833	PC (22:6e/18:4)	C_48_H_78_NO_7_P	7.68	812.56	−1.7959	0.0428	Down
C835	PC (3:0/16:2)	C_27_H_50_NO_8_P	7.41	548.33	−1.4748	0.0265	Down
C85	16-Hydroxyhexadecanoic acid	C_16_H_32_O_3_	8.55	271.23	−2.1233	0.0002	Down
C86	17beta-Trenbolone	C_18_H_22_O_2_	0.57	271.17	−1.8056	0.0048	Down
C862	PC(O-16:0/18:2(9Z,12Z))	C_42_H_82_NO_7_P	0.52	744.59	−2.7272	0.0160	Down
C903	Piperine	C_17_H_19_NO_3_	6.06	286.14	−2.1243	0.0085	Down
C949	SM(d18:0/16:1(9Z)(OH))	C_39_H_77_N_2_O_7_P	8.77	717.55	−2.6979	0.0095	Down
C971	Stercobilin	C_33_H_46_N_4_O_6_	10.18	593.33	−3.0773	0.0235	Down
C100	1-Naphthol	C_10_H_8_O	13.00	145.06	3.6377	0.0085	Up
C1023	YLH	C_21_H_29_N_5_O_5_	11.68	330.22	1.6671	0.0355	Up
C1026	(1,2,3,5,9,18)-1,2,3,19-Tetrahydroxyurs-12-en-28-oic acid	C_30_H_48_O_6_	7.46	505.35	4.5519	0.0156	Up
C1027	(2,3,9,17,22R)-2,3,14,20,22-Pentahydroxyergost-7-en-6-one	C_28_H_46_O_6_	6.28	479.34	5.4252	0.0001	Up
C1036	18--Glycyrrhetinic acid	C_30_H_46_O_4_	9.39	471.35	1.4789	0.0293	Up
C1042	3-O--D-Glucopyranosylandrographolide	C_26_H_40_O_10_	5.82	513.27	2.0579	0.0155	Up
C1044	6-Hydroxy-21-desacetyl deflazacort	C_23_H_29_NO_6_	6.88	416.21	2.3306	0.0235	Up
C1158	lysoPC(26:1(5Z))	C_34_H_68_NO_7_P	8.78	634.48	1.4957	0.0295	Up
C1192	N-morpholino-N-[(5-nitro-3-thienyl)carbonyl]urea	C_10_H_12_N_4_O_5_S	4.01	301.06	4.9909	0.0367	Up
C139	2-Amino-9,10-epoxy-8-oxodecanoic acid	C_13_H_14_N_2_O	13.43	214.11	2.2364	0.0400	Up
C188	3-Hydroxy-cis-5-tetradecenoylcarnitine	C_21_H_39_NO_5_	8.61	386.29	1.7023	0.0219	Up
C223	4-Anilino-4-oxobutanoic acid	C_10_H_11_NO_3_	1.45	194.08	1.4964	0.0461	Up
C229	4-methoxy-6-(prop-2-en-1-yl)-2H-1,3-benzodioxole	C_11_H_12_O_3_	4.16	193.09	3.9123	0.0054	Up
C253	5-Methoxyindoleacetic acid	C_11_H_11_NO_3_	4.06	204.07	4.2022	0.0350	Up
C308	ACar 20:5	C_27_H_44_NO_4_	8.86	447.33	2.6964	0.0272	Up
C318	Actrarit	C_10_H_11_NO_3_	1.03	194.08	1.7087	0.0255	Up
C320	Adenine	C_5_H_5_N_5_	0.60	134.05	1.4160	0.0478	Up
C418	Dehydrocholic acid	C_24_H_34_O_5_	4.75	403.25	3.0042	0.0166	Up
C443	DL-Arginine	C_6_H_14_N_4_O_2_	0.55	175.12	2.7666	0.0202	Up
C542	Hyodeoxycholic acid	C_24_H_40_O_4_	7.95	393.30	3.1296	0.0361	Up
C563	L-Arginine	C_6_H_14_N_4_O_2_	9.18	175.12	2.7596	0.0186	Up
C614	LPE 22:5	C_27_H_46_NO_7_P	7.06	528.31	1.8620	0.0413	Up
C622	LSD-d3	C_20_H_22_D_3_N_3_O	4.02	327.23	3.4250	0.0121	Up
C660	LysoPE(0:0/22:5(4Z,7Z,10Z,13Z,16Z))	C_27_H_46_NO_7_P	6.25	528.31	1.9982	0.0123	Up
C820	PC (18:5e/2:0)	C_28_H_48_NO_7_P	6.96	542.32	1.3976	0.0331	Up
C858	PC(20:1(11Z)/14:1(9Z))	C_42_H_80_NO_8_P	13.25	758.57	1.4247	0.0301	Up
C928	RMK	C_17_H_35_N_7_O_4_S	7.87	434.25	7.8480	0.0001	Up
C942	SM (d23:2/13:0)	C_41_H_81_N_2_O_6_P	10.50	729.59	2.6217	0.0151	Up

## Data Availability

The original contributions presented in this study are included in the article. Further inquiries can be directed to the corresponding authors.

## References

[1] Gao X., Fan Y., Dai K., Zheng G., Jia X., Han B., Xu B., Ji H. (2024). Structural characterization of an acid-extracted polysaccharide from *Suillus luteus* and the regulatory effects on intestinal flora metabolism in tumor-bearing mice. Int. J. Biol. Macromol..

[2] Yin S., Zhang X., Xie J., Ye X., Zhang X. (2023). Change of microbial communities in heavy metals-contaminated rhizosphere soil with ectomycorrhizal fungi *Suillus luteus* inoculation. Appl. Soil Ecol..

[3] Zocher A.-L., Kraemer D., Merschel G., Bau M. (2018). Distribution of major and trace elements in the bolete mushroom *Suillus luteus* and the bioavailability of rare earth elements. Chem. Geol..

[4] Jaworska G., Pogoń K., Bernaś E., Skrzypczak A., Kapusta I. (2014). Vitamins, phenolics and antioxidant activity of culinary prepared *Suillus luteus* (L.) Roussel mushroom. LWT Food Sci. Technol..

[5] dos Santos T., Oliveira M., Sousa D., Lima R.T., Martins A., Ferreira I.C.F.R., Vasconcelos M.H. (2014). *Suillus luteus* methanolic extract inhibits proliferation and increases expression of p-H2A.X in a non-small cell lung cancer cell line. J. Funct. Foods.

[6] Li J., Hu W., Li C., Kuang H., Wang M. (2026). Polysaccharides from *Polygonum multiflorum* Thunb. and its processed products: A review on traditional uses, extraction, structural characteristics, pharmacological activities, structure-activity relationships, and drug delivery applications. Fitoterapia.

[7] Yang J., Lei K., Zhang W., Hu H., Dai M., Zheng Y., Ding W., Yu J. (2026). Structure-activity relationship of *Chroogomphis rutilus* polysaccharides on gut microbiota mediated immunomodulatory effects. Food Biosci..

[8] Sao C.-H., Shih Y.-C., Lin S.-Y., Chang Y.-H., Yang A.C., Chen Y.-J. (2026). Real-time intraoperative identification of malignant ovarian tumors using deep learning of clinical gross specimen images. Taiwan. J. Obstet. Gynecol..

[9] Jiang Y., Gong W., Xian Z., Xu W., Hu J., Ma Z., Dong H., Lin C., Fu S., Chen X. (2023). 16S full-length gene sequencing analysis of intestinal flora in breast cancer patients in Hainan Province. Mol. Cell. Probes.

[10] Hang Z., Lei T., Zeng Z., Cai S., Bi W., Du H. (2022). Composition of intestinal flora affects the risk relationship between Alzheimer’s disease/Parkinson’s disease and cancer. Biomed. Pharmacother..

[11] Khosrojerdi M., Azad F.J., Haghbin A., Azimian A. (2026). Gut microbiota and the immune system: A regulatory axis through host MicroRNA modulation. Infect. Genet. Evol..

[12] Song K., Wu J., Zhen X., Zhu R., Li J., Han P. (2026). Programmable biomanufacturing of fungal and microalgal bioactive polysaccharides: Deciphering the structure-activity mechanisms and establishing rational regulation strategies. Trends Food Sci. Technol..

[13] Ajith D., Kumar S., Chowdhury A.R., Mishra R., Sravani C., Ram M.K., Bishi S.K., Lohot V.D., Tribhuvan K.U. (2026). Prebiotic efficacy of natural gums and their partial hydrolysates: Linking fermentation dynamics with glycosyl hydrolase expression. Food Hydrocoll. Health.

[14] Chen Y., Gong G., Yan L., Zhu Y., Yang J., Zhang X. (2025). Tartary buckwheat soluble dietary fiber: Structure-dependent prebiotic activity and metabolic regulation of gut microbiota. LWT.

[15] Ji H.-F., Li M., Han X., Fan Y.-T., Yang J.-J., Long Y., Yu J., Ji H.-Y. (2025). Lactobacilli-Mediated Regulation of the Microbial–Immune Axis: A Review of Key Mechanisms, Influencing Factors, and Application Prospects. Foods.

[16] Dong X., Sun S., Wang X., Yu H., Dai K., Jiao J., Peng C., Ji H., Peng L. (2024). Structural characteristics and intestinal flora metabolism mediated immunoregulatory effects of *Lactarius deliciosus* polysaccharide. Int. J. Biol. Macromol..

[17] Zhang S., Han J., Gao R., Xu Y., Feng Z., Huang X., Zhong Y., Wang Q., Yu X., Chang S. (2026). Interpretable machine learning for optimization of ultrasound-assisted polysaccharide extraction using natural deep eutectic solvents: From yield enhancement to structural and antioxidant activity validation. Bioresour. Technol..

[18] Dong X., Sun S., Jiao J., Ji H., Jia X., Silviya L.R., Wang X., Liang X., Peng C., Liu A. (2025). Structural characterization and gut microbiota-mediated immunomodulatory effects of a novel fructose enriched polysaccharide from *Platycodon grandiflorus*. Food Res. Int..

[19] Nawrocka A., Krekora M., Niewiadomski Z., Miś A. (2018). FTIR studies of gluten matrix dehydration after fibre polysaccharide addition. Food Chem..

[20] Li Z., Zhang Z., Goksen G., Khan M.R., Ahmad N., Zhang W., Deng H., Zhu K. (2026). Changes in the nanostructure of pectin and cell wall polysaccharides during postharvest softening of wax apple fruit. (*Syzygium Samarangense*). Postharvest Biol. Technol..

[21] Xu L., Yao J., Wang Z., Xie S., Ou J., Wang B. (2025). A methylation-assisted gas chromatography-mass spectrometry strategy for comprehensive monosaccharide profiling in *Polygonatum* polysaccharides: Overcoming limitations of conventional chromatographic techniques. J. Chromatogr. A.

[22] Ji H., Li M., Wang R., Mao D., Ji Z., Peng L., Ding W., Ji H. (2026). Study on the Enrichment Effect of *Suillus luteus* Polysaccharide on Intestinal Probiotics and the Immunomodulatory Activity. Microorganisms.

[23] Gupta A., Cooper V.S., Zemke A.C. (2025). Evaluation of V3–V4 and FL-16S rRNA amplicon sequencing approach for microbiota community analysis of tracheostomy aspirates. mSphere.

[24] Parralejo-Sanz S., Requena T., Cano M.P. (2025). Biotransformation of *Opuntia stricta* var. *dillenii* extracts by human gut microbiota: Evolution of betalains and phenolic compounds, and formation of secondary metabolites. Food Biosci..

[25] Chen Z., Chen H., Ye X., Zhao P., Wang Y. (2025). Study on extraction technology and antioxidant activity of “Sibai” extracts based on orthogonal design and Caenorhabditis elegans. J. Holist. Integr. Pharm..

[26] Chen G., Ran X., Yang Z., Yang X., Li W., Zhao Y., Ma K., Zheng X., Wang A., Jia J. (2026). Structural characterization and anti-aging activity of two polysaccharides from *Trillium tschonoskii*: Insights into their structure—Activity relationships. Int. J. Biol. Macromol..

[27] Ji H., Li M., Mao D., Chen Y., Han B., Zheng Y., Ding W., Ji H. (2026). Structural Characteristics and Gut Microbiota-Mediated Immunomodulatory Mechanisms of Water- and Alkali-Extracted Polysaccharides from *Tuber indicum*. Nutrients.

[28] Er M., Orakdogen N. (2024). Bioactive interpenetrating hybrids of poly(hydroxyethyl methacrylate-co-glycidyl methacrylate): Effect of polysaccharide types on structural peculiarities and multifunctionality. Int. J. Biol. Macromol..

[29] Nan Z., Chen L., Li G., Li H., Li Y., Ma J., Ding J., Yang J. (2025). A method for the quantitative analysis of *Lycium barbarum* polysaccharides (LBPs) using Fourier-transform infrared spectroscopy (FTIR): From theoretical computation to experimental application. Spectrochim. Acta Part A Mol. Biomol. Spectrosc..

[30] Yang M., Li X., Du X., Li F., Wang T., Gao Y., Liu J., Luo X., Guo X., Tang Z. (2025). Ultrasound-Assisted extraction and purification of polysaccharides from *Boschniakia rossica*: Structural Characterization and antioxidant potential. Ultrason. Sonochemistry.

[31] Wu Y., Song Y., Li R., Han Z., Li L., Yan Y. (2025). The protective effect of two highly branched polysaccharides from corn silk fermented by *Lactobacillus plantarum* against acute liver injury. Carbohydr. Polym..

[32] Dai K.-y., Ding W.-j., Li Z.-t., Liu C., Ji H.-y., Liu A.-j. (2024). Comparison of structural characteristics and anti-tumor activity of two alkali extracted peach gum arabinogalactan. Int. J. Biol. Macromol..

[33] Fajar J.K., Rizqiansyah C.Y., Runtuk K.S., Purborisanti D.K., Radhiyah R., Pawestri A.R., Sulistomo H.W., Samsu N. (2026). Gut microbiota alpha diversity in hemodialysis patients versus controls: A meta-analysis of chao1, shannon index, and observed species. Clin. Epidemiol. Glob. Health.

[34] Yi G., Kim T., Lee J., Yoon S.J., Lee C., Yoo Y., Lee S.H., Kim J.-J., Kwon B.-O., Hong S. (2026). eDNA-based microbial communities enable rapid screening of sediment pollution. Mar. Pollut. Bull..

[35] Gong Y., Guo N., Dai K., Han B., Wang Z., Ji H. (2025). Primary structure analysis of cold water-soluble alcohol extract from green tea and the regulatory effects on intestinal flora metabolism. LWT.

[36] Li Y., Zhang J., Li Y., Su Y., Li N. (2026). Short-chain fatty acids attenuate obesity-induced oxidative stress by regulating the MUFA/PUFA ratio in phospholipids. J. Funct. Foods.

[37] Aminizadeh S., Zarezadehmehrizi A., Amiri Deh-Ahmadi M., Shahouzehi B. (2026). Role of L-carnitine in exercise training: Anti-inflammatory, antioxidant, and metabolic interactions. Adv. Redox Res..

[38] Jagot F., Gaston-Breton R., Choi A.J., Pascal M., Bourhy L., Dorado-Doncel R., Conzelmann K.-K., Lledo P.-M., Lepousez G., Eberl G. (2023). The parabrachial nucleus elicits a vigorous corticosterone feedback response to the pro-inflammatory cytokine IL-1β. Neuron.

[39] Wang H., Jia Y., Feng T., An B., Ma H., Ren X., Zhang N., Li F., Wei Q. (2024). Development of reusable electrochemiluminescence sensing microchip for detection of vomitoxin. Talanta.

[40] Song S.-J., Wang L., Li L., Bai S.-H., Zhu X.-L., Zhang X.-L., Deng C.-H., Han Z.-G. (2026). Arid1a deficiency promotes metabolic dysfunction-associated steatohepatitis and hepatocellular carcinoma by disrupting the FXR-gut microbiota-bile acid axis. Cancer Lett..

[41] Meza-Perez S., Liu M., Silva-Sanchez A., Morrow C.D., Eipers P.G., Lefkowitz E.J., Ptacek T., Scharer C.D., Rosenberg A.F., Hill D.D. (2024). Proteobacteria impair anti-tumor immunity in the omentum by consuming arginine. Cell Host Microbe.

[42] Salimova E.V., Parfenova L.V., Ishmetova D.V., Zainullina L.F., Vakhitova Y.V. (2023). Synthesis of fusidane triterpenoid Mannich bases as potential antibacterial and antitumor agents. Nat. Prod. Res..

[43] Xiong X., Zhu X., Yu D., Huang Q., Wu X., Tan G., Sun P. (2025). Piperidine polyketide alkaloids of bacterial origin: Occurrence, bioactivity, and biosynthesis. Eur. J. Med. Chem..

[44] Alam F., Malik S. (2026). Bioactive alkaloids as phytotherapeutic agents from family Rutaceae. Pharmacol. Res. Nat. Prod..

[45] Subramani E., Pilié P.G., Slack-Tidwell R., Viscuse P.V., Kuang X., Kandasamy T., Awad D., Han J.J., Huang L., Peterson C.B. (2026). Targeting arginine metabolism overcomes chemotherapy resistance in aggressive-variant prostate cancers. iScience.

[46] Fan Y., Ji H., Han X., Dong X., Ding W., Yu J., Peng L., Ji H. (2025). Structural characteristics and intestinal immunity of *Codonopsis pilosula* polysaccharides prepared by electrolytic water. Ind. Crops Prod..

[47] Chen X., Dai P., Zhu Y., Jin W., Xie Y., Yang Z., Wang Z., Jin X., Lei M., Shi L. (2026). A triple-helix white tea polysaccharide alleviates ulcerative colitis via the gut microbiota–metabolites–inflammation regulatory axis. Carbohydr. Polym..

[48] An Y., You Q., Wang B., Shi Y., Han C., Liu J. (2026). Polysaccharide–gut microbiota interactions in metabolic diseases: Structural selectivity, mediating mechanisms, and evidence deficiencies: A review. Int. J. Biol. Macromol..

[49] Saikia T., Das S., Rajak P., Kalita J.M. (2026). From fermentation to pharmabiotics: Engineering kefir-derived *Lactobacillus* for targeted intratumoral immunotherapy in colorectal Cancer. Int. Immunopharmacol..

[50] Mazumder S., Bhattacharya D., Lahiri D., Nag M., Maity S., Saha D., Kumar N., Singh V., Sharma S., Dwivedi S.P. (2026). Food-derived dietary alkaloids: Structure–biofunctionality relationships in modulating gut microbial biofilms for downregulation of colorectal carcinogenesis. Food Res. Int..

[51] Maeta S., Fukunaga A., Egashira Y., Akahori Y., Wang L., Seo N., Atarashi Y., Ejima D., Fujiwara H., Shiku H. (2026). Introducing arginine residues into scFv in CAR-T cells shows promising antitumor effects. Mol. Ther..

